# Five actionable pillars to engage the next generation of leaders in the co-design of transformative ocean solutions

**DOI:** 10.1371/journal.pbio.3001832

**Published:** 2022-10-17

**Authors:** Erin V. Satterthwaite, Valeriya Komyakova, Natalia G. Erazo, Louise Gammage, Gabriel A. Juma, Rachel Kelly, Daniel Kleinman, Delphine Lobelle, Rachel Sapery James, Norlaila Binti Mohd Zanuri

**Affiliations:** 1 California Sea Grant, Scripps Institution of Oceanography, University of California, San Diego, California, United States of America; 2 Institute for Marine and Antarctic Studies, University of Tasmania, Hobart, Tasmania, Australia; 3 Centre for Marine Socioecology, University of Tasmania, Hobart, Tasmania, Australia; 4 Scripps Institution of Oceanography, University of California, San Diego, California, United States of America; 5 Department of Biological Sciences and Marine & Antarctic Research for Innovation & Sustainability (MARIS), University of Cape Town, Cape Town, South Africa; 6 Alfred Wegener Institute, Helmholtz Centre for Polar and Marine Research, Biologische Anstalt Helgoland, Helgoland, Germany; 7 Seaworthy Collective, Miami, Florida, United States of America; 8 Institute of Marine and Atmospheric Research, Utrecht University, Utrecht, Netherlands; 9 Blue Pacific Programs Manager, WWF-Australia, Gubbi Gubbi Country, Sunshine Coast; 10 Centre for Marine and Coastal Studies (CEMACS), Universiti Sains Malaysia, Gelugor, Pulau Pinang, Malaysia; Brown University, UNITED STATES

## Abstract

Solutions to complex and unprecedented global challenges are urgently needed. Overcoming these challenges requires input and innovative solutions from all experts, including Early Career Ocean Professionals (ECOPs). To achieve diverse inclusion from ECOPs, fundamental changes must occur at all levels—from individuals to organizations. Drawing on insights from across the globe, we propose 5 actionable pillars that support the engagement of ECOPs in co-design processes that address ocean sustainability: sharing knowledge through networks and mentorship, providing cross-boundary training and opportunities, incentivizing and celebrating knowledge co-design, creating inclusive and participatory governance structures, and catalyzing culture change for inclusivity. Foundational to all actions are the cross-cutting principles of justice, equity, diversity, and inclusivity. In addition, the pillars are cross-boundary in nature, including collaboration and innovation across sectors, disciplines, regions, generations, and backgrounds. Together, these recommendations provide an actionable and iterative path toward inclusive engagement and intergenerational exchange that can develop ocean solutions for a sustainable future.

## Abstract 2

Early Career Ocean Professionals (ECOPs) need to engage in co-design processes that address ocean sustainability. This Consensus View proposes five pillars to provide an actionable and iterative path toward inclusive engagement and intergenerational exchange that can develop ocean solutions for a sustainable future.

## Introduction

Marine environments provide a plethora of ecosystem services: from carbon sequestration and oxygen production, to resource provision for human consumption and transport, and they are home to over 2 million eukaryotic species [1–5]. Once presumed highly resilient, these vital environments are experiencing intensifying deleterious impacts as a result of increasing anthropogenic activity [6–8]. To add to historical impacts such as overexploitation and chemical pollution [6,9,10], today’s marine ecosystems also face emerging impacts such as climate change, including marine heatwaves [11,12] and extreme weather events [13], the expansion of marine construction [14], and novel pollution including microplastics, noise, and light [15].

The range of impacts and difficulties associated with marine management render ocean sustainability a “wicked problem” [16]. This problem demands transformative, transdisciplinary approaches, if foundational changes in our complex socioecological systems are to be achieved [17,18]. Such transdisciplinary approaches look beyond single sectors and disciplines, and include co-designing and co-producing research and knowledge in equitable, transparent ways, and using tools and processes that develop a shared commitment and understanding among diverse stakeholders [19]. For example, co-produced research is one way to generate knowledge that can more effectively contribute to sustainability transformations [18,20]. Here, we define sustainability co-production as an “iterative and collaborative process involving diverse types of expertise, knowledge, and actors to produce context-specific knowledge and pathways towards a sustainable future” [18].

Developing transformative solutions requires working with and among a diversity of perspectives, including across career stages, generations, sectors, disciplines, and regions, to generate innovative and sustainable ideas [21]. Early career ocean professionals (ECOPs; Box 1), the voice and focus of this paper, can bridge generational gaps, enhance knowledge transfer, and develop innovative solutions to novel problems [22].

**Box 1. What is an Early Career Ocean Professional?**

The term Early Career Ocean Professional (ECOP) includes individuals working in ocean research and/or practice, including, but not limited to: academia, industry, consultancy, foundations, non-profit organizations, and government positions who identify as being early in their career. However, a universal definition of “early career” is challenging since definitions vary across institutions. In many cases, definitions are related to criteria such as: years since terminal degree, years of professional experience, and/or age. Since what is considered “early career” is context dependent, we do not provide a rigid, specific definition of the term, as one does not exist. Instead, our goal in using this term is to be inclusive of professionals that are near the beginning of their career trajectory.

The aim of this Consensus View is to outline how to engage the next generation of ocean professionals in the co-design of ocean solutions. ECOPs today face many interpersonal, institutional, and cultural challenges to engagement, including limited knowledge-sharing and mentorship opportunities, and limited access to training that targets essential skills for working across sectors and disciplines [23,24]. In addition, access to professional opportunities, support, and funding to develop networks and to participate in transdisciplinary, interdisciplinary, and cross-sector projects is often scarce [25]. Further, existing and traditional metrics of “success” frequently do not align with co-design goals and may fail to recognize other achievements and contributions outside of research (e.g., mentorship, teaching, community engagement; [26,27]), hindering ECOPs professional growth. The current workplace culture, including the casualization of the workforce and dominance of short-term, insecure contracts [24,25], the prevalence of unpaid positions including internships [28], unequitable and unbalanced workloads across the sector [29], as well as documented bullying [30] have led to reduced retention rates of ECOPs, including within ocean science and sustainability fields, and in particular for marginalized groups [31].

These challenges are further exacerbated for underrepresented, marginalized, and disadvantaged groups, including women [32], people of color, Indigenous Peoples, individuals from developing nations, people with disabilities, and LGBTQIA+ representatives [33–35]. For example, ecological research remains largely dominated by exclusionary Western approaches [36]. This is despite extensive evidence across multiple fields that diverse teams are more innovative and productive [34,37–39]. In addition, ECOPs from underrepresented communities often experience the intersection of many forms of discrimination, so may face cumulative effects of different barriers [40]. In this Consensus View, we acknowledge that these groups face additional barriers that include, but are not limited to, power imbalances, limited access to social and physical capital, and continued marginalization. Addressing this added layer of complexity is beyond the scope of this paper given the context-specific sensitivity that is required in building inclusivity in our diverse spaces and points of departure.

It is apparent that many barriers, biases, and challenges hinder inclusion and collaboration of early career ocean professionals in the co-design of sustainable ocean solutions [25,26,41–44]. Given these barriers and challenges, guidance on how to better engage ECOPs in the co-design of transformative ocean solutions is vitally needed [45]. Today, some frameworks and pathways to address such challenges have been proposed in certain fields, including polar research [43], and within the wider marine research sector [46]. In addition, international processes, such as the United Nations Decade of Ocean Science for Sustainable Development (2021–2030), hereafter referred to as the UN Ocean Decade [47], are working to provide ECOPs with professional opportunities to engage in international ocean sustainability initiatives [48,49].

In this Consensus View, we identify and explore solutions for improving the engagement of ECOPs in co-design processes in support of sustainability. We, the authors, are a cohort of ECOPs with diverse practical experiences and expertise spanning different sectors (including research, industry, NGOs, and government) and multiple academic disciplines (including marine biology, oceanography, socioecology, ecology, governance, and marine sustainability). Collectively, we span 6 regions of the world, including Oceania, North and South America, Africa, Asia, and Europe; however, we do not purport to be representative of the entire global ECOP community. Rather, our intent here is to present prospective thinking and to develop ongoing discussions on how ECOPs can be championed and engaged in shaping transformative ocean solutions for a sustainable future.

## The 5 actionable pillars

**Fig 1:**
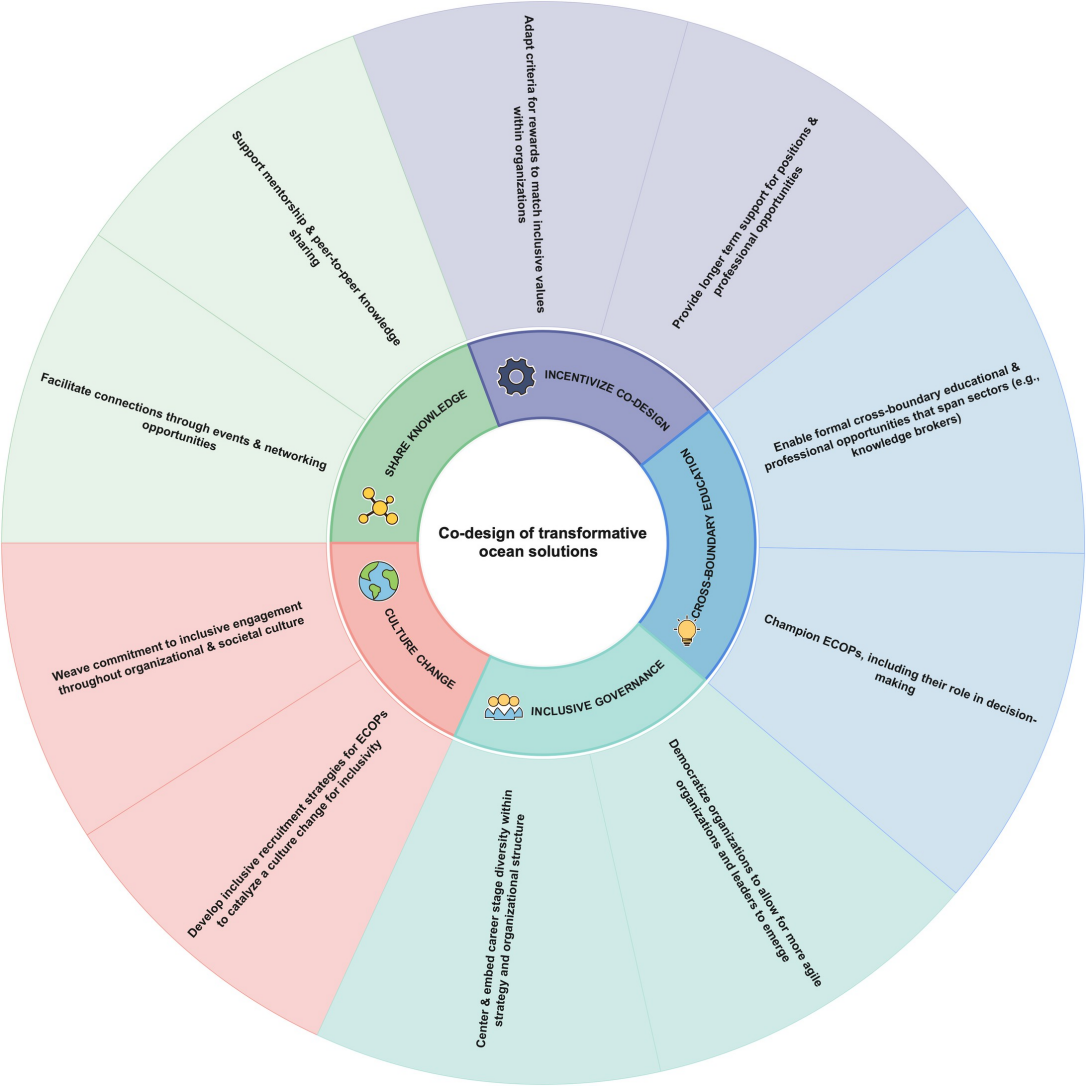
Five actionable pillars to engage the next generation of ocean leaders in the co-design of transformative ocean solutions. Solutions include those that can be taken by individuals within their professional relationships, networks, institutions, and society. The figure was created with EdrawMind, a conceptual chart mapping software.

We present 5 actionable pillars that individuals can take within their interpersonal relationships, institutions, and professional networks: 1. share knowledge, 2. provide cross-boundary training and opportunities, 3. incentivize and celebrate knowledge co-design, 4. create inclusive governance structures, and 5. catalyze culture change for inclusivity (Fig 1). Most of the proposed actions can largely happen independent of each other, although would have a much greater impact together. Foundational to all actionable pillars are the cross-cutting principles of justice, equity, diversity, and inclusivity. In addition, the actions are cross-boundary in nature, including collaboration and innovation across sectors, disciplines, regions, generations, and backgrounds. Together, these recommendations provide an actionable and iterative path toward inclusive engagement and intergenerational exchange.

### 1. Share knowledge through networks and mentorship

Knowledge creation and transferal is the result of collaborative processes whereby people share and combine expertise, information, and resources in new ways to create valuable and novel solutions [50]. Building trust and sharing skills, knowledge, and power are foundational to such processes, including knowledge co-design [18]. However, due to their early career stage, ECOPs are generally limited in their opportunities to build strong networks and relationships [46]. If the next generation of ocean researchers and leaders are to be successful in their aims to co-design solutions for ocean sustainability, then creating more meaningful opportunities for ECOPs to engage in relationship-building, knowledge sharing, and effective collaboration is key.

#### Facilitate connections through events and networking opportunities

Developing and providing explicit networking opportunities, whether through informal or formal mechanisms, can help ECOPs find and connect with stakeholders and other professionals outside their field of expertise. Professional networking opportunities can include in-person or virtual events such as research and stakeholder workshops or conferences, as well as more informal gatherings (e.g., socializing over drinks or a meal) that can be a way for ECOPs to be more comfortable during networking. Virtual networking can also be supported via social media or online communication platforms [51]. For example, virtual workshops, such as the Virtual ECOP Day (June 2021; a workshop to enhance ECOPs network building and knowledge sharing) and The Future of Ocean Plastic (April 2022; a workshop to establish guidelines for stakeholder engagement in the ocean plastics field), facilitated networking, knowledge exchange, and co-creation of science between ECOPs, experienced ocean professionals, and a diverse array of stakeholders. In fact, many of the co-authors of this paper first met and built relationships through the Ocean Visions Summit 2021, which led to this collaborative work.

In order to be more inclusive, events and networking opportunities must be tailored to the community of interest and developed collaboratively. For example, inviting marginalized voices into networks or spaces not built for them or that undermine their expertise or live experiences can often work against the goals of inclusion [52]. Thus, ECOPs that self-identify as marginalized can join organizations that promote minority groups to engage in these conversations. For example, the Society for Advancement of Chicanos/Hispanics and Native Americans in Science (SACNAS), South Africa’s Black Women in Science (BWIS) groups, and Black Women in Ecology, Evolution, and Marine Science (BWEEMS) are a few examples of societies that serve as essential access points and networks for ECOPs from historically disadvantaged groups.

#### Support mentorship and peer-to-peer knowledge sharing

Mentors, especially senior and experienced professionals, can share knowledge, champion and provide opportunities for ECOPs to become agents of change and influence [53]. Formalized mentorship programs, especially those that are inclusive of researchers and non-academic practitioners (e.g., managers, consultants), can support ECOPs in developing their own professional relationships and networks: for example, through professional societies and associated conferences (e.g., AGU Mentoring 365), communities of practice, and fellowship programs [54]. Establishing and connecting networks of researchers and practitioners can also support others to build trust, engage with different knowledge systems, and better contextualize problems and solutions [18].

Knowledge sharing and learning can also occur at peer-to-peer levels [55]: for example, via the Ocean Decade ECOP Programme, of which we are a part [49], and other intergenerational networks that are also emerging, including the OceanBRIDGES (Bridging Ocean Research, Innovation, and Diversity across Generations of Experts and Stakeholders). OceanBRIDGES organizes 2-way knowledge-sharing events, ECOPs are provided with a platform to share their expertise and act as mentors for experienced ocean professionals (e.g., sharing their knowledge about technology and communications).

### 2. Provide cross-boundary education and opportunities

ECOPs also need training and support to guide their efforts in cultivating knowledge related to cross-sector, transdisciplinary, and diverse engagement, including entrepreneurial work [56]. However, despite a growing number of cross- and interdisciplinary graduate programs, single disciplinary training remains the norm, often as a result of the traditionally siloed nature of university departments [57]. ECOP training opportunities should also be complemented by professional development opportunities if ECOPs are to effectively participate in and contribute to co-design processes for sustainability. These opportunities may include, for example, experiences in knowledge brokering (i.e., transferring information between disciplines, organizations, and sectors) [58] and championing ECOPs in decision-making bodies, to equip and empower them for leadership positions.

#### Enable formal cross-boundary educational opportunities

Formal education systems involve curriculum-based learning in education and research institutions (e.g., universities), which house most ECOPs at some point in their career, with many programs being highly specialized within a single field. Today, there is growing demand for interdisciplinary training [54], where ECOPs work across cognate fields within and across social and natural science realms [59–61]. It also involves collaborating and exchanging knowledge across policy, science, and practitioner divides [62]. This means that training should include core non-disciplinary skills, such as team development, project management, and soft skills, such as communication and problem-solving [23,57,63]. Some programs have started to incorporate a wider array of skills and competencies into their curriculums. Examples include the Marine and Antarctic Science Master’s Program at the Institute for Marine and Antarctic Studies (IMAS), where several units target transferable skills, such as project management and stakeholder engagement, and the Program for Interdisciplinary Environmental Research at the University of California, San Diego, which is focused on developing solutions to critical environmental problems and has cross-sector capstone projects.

#### Develop professional opportunities that span sectors (e.g., knowledge brokers)

Boundary-spanning work requires developing partnerships between diverse groups, organizations, sectors, and regions [64]. ECOPs can play instrumental roles in connecting diverse and disparate groups and sectors. For example, by working as knowledge brokers—people positioned between different interested parties (researchers, communities, other end-users) to facilitate knowledge co-production and mobilization—ECOPs can facilitate the sharing and uptake of new knowledge across complex organizational research ecosystems, industries, and communities to deliver value. For example, the California Sea Grant Extension Program provides ECOPs with opportunities to work with academic and government agencies across state and federal boundaries, and to gain unique experiential knowledge. In addition, cross-sector pipelines, such as those from academia to entrepreneurship, offer an important pathway to develop ECOPs’ capacity to contribute to sustainability solutions [65]. Even with extensive theoretical training, meaningful hands-on practice and reflection offers experience on how co-design is actually carried out in diverse contexts, with different partners, and within unique situations [66,67]. Hands-on experiences can include paid sustainability internships and fellowships that explicitly focus on co-design.

#### Champion ECOPs, including their role in decision-making

Giving ECOPs a seat at the table in leadership bodies, steering committees, and advisory groups can help to ensure that decision-making, leadership agendas, and future-looking strategies are co-created with emerging voices and perspectives and facilitate a sense of collective ownership in how sustainable ocean futures are shaped. Critically, however, these types of dialogues must be an inclusive fora where ECOPs are valued for their unique expertise and perspectives. Organizations at all levels, from local to global scales, can provide ECOPs with a platform to let their voices be heard and to contribute to intergenerational knowledge transfer and learning. For example, career stage diversity is increasingly included in high-level committees and organizational bodies within international policy processes (e.g., the UN Ocean Decade), professional societies (e.g., The Oceanography Society), international science organizations (e.g., PICES, GEOBlue Planet, Ocean Visions Global Ecosystem for Ocean Solutions Program), and NGOs (e.g., Pacific Regional Environment Program). Further, many funding bodies are now requiring diverse participation in projects: including the National Research Foundation (NRF) in South Africa, who champion early career researchers and minority groups (including people of color, women, and people with disabilities) in projects, by prioritizing them through the grant award scoring system.

### 3. Incentivize and celebrate knowledge co-design

To date, engagement in co-design and collaborative transdisciplinary work is poorly recognized and rewarded. As such, we see a need to incentivize and reward knowledge co-design efforts for transformative ocean solutions [68]. This is especially important in the context of ECOPs, who are often the most dependent on traditional metrics and evaluation systems for career progression. The misalignment of these metrics toward engagement and co-design can discourage emerging, as well as more senior, researchers to pursue these avenues of work [69]. Incentives can be delivered through more formal (organizational) reward systems and recognition including awards and fellowships, and/or via informal (interpersonal) ways, such as positive relationship building and recognition from peers, supervisors, or other personal and professional connections.

#### Adapt criteria for rewards to match inclusive values within organizations

Formal and organizational recognition often occurs through rewards and merit reviews. In order to incentivize diverse and inclusive co-design, recognition and advancement should reflect values associated with co-design and cross-boundary work, including the quality, impact, and open science components of the work. For example, if collaboration is a core value, merit reviews should also value demonstrating positive teamwork, global networking, and providing open science, rather than individual performance as is the norm. Similarly, core metrics of success can be broadened to value demonstrated mentorship, leadership, diversity, inclusion, and research team well-being [27]. Additional metrics could include the uptake of knowledge into decision-making processes and on its further impacts on individuals, communities, organizations, and political processes such as related to relationships, trust, changes in attitude or behavior, and mutual learning [46]. Furthermore, to guide diverse ECOPs with different approaches and backgrounds, career planning tools such as Individual Development Plans [70] can be used to allow for various metrics to be included and evaluated. To complement this, training programs for supervisors of ECOPs could be included to ensure that they have the right skills to evaluate successes of ECOPs from this new perspective. These tools have the potential to provide a tailored, holistic, and impact-centered merit review and are beneficial for underrepresented ECOPs that may have skills that are outside of the conventional review schemes.

For example, some programs like Utrecht University in the Netherlands are using broader criteria for evaluation. Specifically, during annual reviews researchers are assessed and rewarded based on the societal and decision-making impact, public engagement, teaching, and trans-disciplinary and cross-sectoral elements of collaborative projects.

#### Provide longer term support for positions and professional opportunities

Co-design processes often take extensive time [65]. However, the current academic employment model is typified by short-term contracts [25,26,28]. These insecure employment conditions have personal impacts on ECOPs, including stress and resulting impacts on mental health [71], as well as broader consequences, including reduced commitment to the institutions, unfinished projects resulting from insufficient time within these short-term contracts, or time spent elsewhere on necessary new employment searches and job applications [72,73]. A much needed and much called for system change from short-term temporary contracts to more permanent positions can support and better retain ECOPs in marine-related disciplines. Similarly, remote positions can help to retain ECOPs, since it allows ECOPs with strong support networks to remain in their local communities. Some organizations have been implementing positive changes to work toward tackling these challenges, such as the University of Tasmania, Australia Pathways Program that was implemented nearly 5 years ago with the goal of providing pathways to ongoing employment after 7 years of short-term employment with the University.

### 4. Create inclusive and participatory governance structures

Providing leadership opportunities for ECOPs to engage in knowledge co-design, requires that there are governance structures within institutions and organizations focused on co-design to support inclusive engagement. Since organizations are one of the primary mechanisms through which inequities are maintained in society, this can be achieved through democratizing organizations [74] to allow for more agile organizations and leaders to emerge [75] as well as centering career stage diversity in organizational structure.

#### Democratize organizations to allow for more agile organizations and leaders to emerge

Moving away from typical traditional hierarchy models of organizational governance to more democratic organizations can promote inclusion, transparency, shared decision-making, as well as organizational agility [74]. More participatory organizational models catalyze a culture of accountability and shared leadership at all levels [75]. As such, more democratic organizations that adopt codes of conduct and action plans to support marginalized ECOPs provide a mechanism for more diverse ECOP champions and leaders to emerge.

For example, some institutions are decentralizing their organizational structures, such as by breaking teams into smaller units, as well as enabling decision-making autonomy at all levels to allow for personal development and experience with responsibility. For example, the Global ECOP Programme was envisioned as a polycentric network to promote widespread participation and cooperation of ECOPs from global to local scales. The Global ECOP Programme has evolved into a group of regional and global networks cooperatively working toward the goal of inclusive engagement within the UN Ocean Decade [49].

#### Center and embed career stage diversity within strategy and organizational structure

In addition to making institutions more democratic, career stage diversity can be embedded into existing organizational structures or new governing bodies can be developed within existing organizations that prioritize and focus on diversity and inclusivity. Diversity and inclusion committees, advisory bodies, and other organizational structures can support strategic diversity leadership and direction [76]. For example, within the context of the UN Ocean Decade a new structure, the Global ECOP Programme, was developed to ensure that career stage diversity is included throughout all Decade actions [49]. Other UN Ocean Decade programs have embedded career stage diversity in their advisory panels, committees, and other governing bodies. In addition, internal and external assessment and audits on employment and promotion practices can ensure that organizations are facilitating inclusive practices and working toward learning and sharing best practices for inclusive engagement [77].

### 5. Catalyze culture change for inclusivity

Addressing complex social and environmental issues can be supported by fostering a culture that can recognize and celebrate different knowledge systems—including intergenerational diversity—to inform decisions and actions that achieve long-term, resilient solutions. As such, a culture that acknowledges and empowers ECOPs to design, implement, and scale sustainable solutions is critical [78].

#### Weave commitment to inclusive engagement throughout organizational and societal culture

Co-design processes need to be built for inclusivity and creativity [65]. Fostering a culture of inclusion requires developing trust, integrity, openness to different standpoints, and integrating different voices into open and participative dialogue [79]. These principles can be embedded into organizations and institutional elements, such as values, strategy, and policies, that can reflect and bolster cultures that support inclusive engagement and co-design related to sustainability [80,81].

For example, the Seaworthy Collective, a startup community and venture studio in the United States of America, focuses on the support and development of entrepreneurs and investors in ocean and climate solutions, especially ECOPs, across different forms of human capital, including intellectural, social, and cultural capital [82]. These efforts support personal development through access to mental health resources, developing methodologies for innovation, and fostering relationship and network building.

Working toward inclusive engagement requires understanding the historical underpinnings of existing structures, building contemporary and future cultural competence, and having humility. Given that ocean science and related disciplines are deeply connected to the histories of colonization and the perpetuation of unequal ocean governance structures for Indigenous Peoples and local communities [36], re-imagination of historical, cultural, and personal understandings are needed to develop the structural changes that can foster equal sharing and recognition of diverse knowledges within existing institutions.

#### Develop inclusive recruitment strategies for ECOPs

Organizational culture affects the types of people that can apply for and are subsequently hired by an organization. Whether being hired to work independently or in a team, ECOPs should be hired not only for the knowledge and experience they bring, but also for the new perspectives they contribute to a workplace [83]. Inclusive recruitment strategies result in organizations that foster creativity and diverse thinking for realizing innovation and agile design that can generate unique and inventive ocean solutions [84]. For example, ECOPs can bring new ideas and ways of thinking to stagnant silos. Different types of ECOPs can be supported and skills leveraged depending on their strengths, interests, and personalities. Roles can include knowledge carriers and retainers, interpreters and sense makers, networkers and facilitators, stewards and leaders, visionaries and experimenters, and followers and reinforcers [85], all of which are important for building adaptive capacity within organizations and supporting emerging leaders.

## Looking ahead

We have demonstrated that a multifaceted approach is needed to facilitate and enhance greater ECOP participation and recognition to effect meaningful change. This paper identified 5 actionable pillars for supporting and championing intergenerational participation in the co-design of transformative ocean solutions (Fig 1). Each of these actions emphasizes the need for cross-boundary and collaborative effort; this is a complex task. We acknowledge that the type of transformative change required will neither happen quickly nor easily; however, we are hopeful that the guidelines we have outlined can bring change-makers closer to attaining their goals.

The examples we presented highlight a range of informal and formalized changes that can occur from interpersonal to organizational levels. Interpersonal professional relationships must move beyond current silos to facilitate better cross-disciplinary and cross-scale knowledge sharing through networking, mentorship, and peer-to-peer knowledge exchange.

Importantly, organizations play a key role in removing barriers to intergenerational participation, and as such, should be central in charting the course for the future. Experienced professionals within ocean sustainability institutions can provide cross-boundary training, professional opportunities, and can ensure that knowledge co-design is incentivized, rewarded, and recognized. In addition, engagement of ECOPs can be facilitated by developing inclusive and participatory governance structures and fostering inclusive cultures.

We recognize that context is critically important; examples should be assessed and adapted to reflect the specific needs of individuals and communities and at the needs of individuals and communities and at the scale that they are applied. Given this, we further emphasize that engagement processes start with relationship building—including sharing and listening to the needs of the contributors and communities and building trust and social license among all actors. Co-design involves working side by side with diverse people, such as from different disciplines and sectors, to address complex issues [86]. The themes and examples presented above detail the myriad of ways that professionals working in environmental, marine, and ocean careers can be supported to engage in solutions in a co-designed, participatory, and inclusive way.

Solutions to complex, unprecedented, and persistent global socioecological challenges are urgently needed. Overcoming these challenges requires input and innovative solutions from all experts, including ECOPs [18,22]. To achieve ECOP inclusion, engagement, and leadership, fundamental changes need to occur at all levels of society. These changes will need to center around values related to transformation for sustainability, including systems and holistic thinking, co-creation, innovation, and adaptation. We highlight that minority groups, including women, people of color, Indigenous Peoples, individuals from developing nations, and LGBTQIA+ representatives, have been typically underrepresented in marine science. If true diversity and inclusivity are to be achieved, then attention to barriers to inclusion and engagement experienced by these groups, especially at early career stages, must be identified and addressed.

Transformative change and adaptation necessitates continuous learning and self-organization [87]. This paper offers prospective thinking and contributes to ongoing discussions on how ECOPs can be championed and engaged in shaping transformative ocean solutions. We hope that others can further develop on the pillars, dialogue, and learning presented here.
